# Impact of COVID-19 Lockdown in Eating Disorders: A Multicentre Collaborative International Study

**DOI:** 10.3390/nu14010100

**Published:** 2021-12-27

**Authors:** Isabel Baenas, Mikel Etxandi, Lucero Munguía, Roser Granero, Gemma Mestre-Bach, Isabel Sánchez, Emilio Ortega, Alba Andreu, Violeta L. Moize, Jose-Manuel Fernández-Real, Francisco J. Tinahones, Carlos Diéguez, Gema Frühbeck, Daniel Le Grange, Kate Tchanturia, Andreas Karwautz, Michael Zeiler, Hartmut Imgart, Annika Zanko, Angela Favaro, Laurence Claes, Ia Shekriladze, Eduardo Serrano-Troncoso, Raquel Cecilia-Costa, Teresa Rangil, Maria Eulalia Loran-Meler, José Soriano-Pacheco, Mar Carceller-Sindreu, Rosa Navarrete, Meritxell Lozano, Raquel Linares, Carlota Gudiol, Jordi Carratala, Maria T. Plana, Montserrat Graell, David González-Parra, José A. Gómez-del Barrio, Ana R. Sepúlveda, Jéssica Sánchez-González, Paulo P. P. Machado, Anders Håkansson, Ferenc Túry, Bea Pászthy, Daniel Stein, Hana Papezová, Jana Gricova, Brigita Bax, Mikhail F. Borisenkov, Sergey V. Popov, Denis G. Gubin, Ivan M. Petrov, Dilara Isakova, Svetlana V. Mustafina, Youl-Ri Kim, Michiko Nakazato, Nathalie Godart, Robert van Voren, Tetiana Ilnytska, Jue Chen, Katie Rowlands, Ulrich Voderholzer, Alessio M. Monteleone, Janet Treasure, Susana Jiménez-Murcia, Fernando Fernández-Aranda

**Affiliations:** 1 Department of Psychiatry, Bellvitge University Hospital-IDIBELL, 08907 Barcelona, Spain; ibaenas@bellvitgehospital.cat (I.B.); mikeletxandi@gmail.com (M.E.); laarcreed_lm@hotmail.com (L.M.); isasanchez@bellvitgehospital.cat (I.S.); jsanchezg@bellvitgehospital.cat (J.S.-G.); 2 CIBER Fisiopatología Obesidad y Nutrición (CIBERobn), Instituto de Salud Carlos III, 28029 Barcelona, Spain; Roser.Granero@uab.cat (R.G.); jmfreal@idibgi.org (J.-M.F.-R.); fjtinahones@uma.es (F.J.T.); carlos.dieguez@usc.es (C.D.); gfruhbeck@unav.es (G.F.); 3 Psychoneurobiology of Eating and Addictive Behaviors Group, Neurosciences Programme, Bellvitge Biomedical Research Institute (IDIBELL), 08908 Barcelona, Spain; 4 Department of Psychobiology and Methodology, School of Psychology, Universitat Autònoma de Barcelona, 08193 Barcelona, Spain; 5 Facultad de Ciencias de la Salud, Universidad Internacional de La Rioja, 26006 La Rioja, Spain; gemma.mestre.bach@gmail.com; 6 Endocrinology and Nutrition Division, Hospital Clinic and Institut d’Investigacions Biomèdiques August Pi Sunyer (IDIBAPS), 08036 Barcelona, Spain; eortega1@clinic.cat (E.O.); aandreu@clinic.cat (A.A.); vmoize@clinic.cat (V.L.M.); 7 Centro de Investigación Biomédica en Red de Diabetes y Enfermedades Metabólicas Asociadas (CIBERDEM), 28029 Madrid, Spain; 8 Unit of Diabetes, Endocrinology and Nutrition, Hospital de Girona Dr. Josep Trueta-Institut d’Investigació Biomèdica de Girona (IDIBGI), 17007 Girona, Spain; 9 Department of Medical Sciences, School of Medicine, University of Girona, 17004 Girona, Spain; 10 Department of Endocrinology and Nutrition, Virgen de la Victoria University Hospital-Instituto de Investigación Biomédica de Málaga (IBIMA), 29010 Málaga, Spain; 11 Department of Physiology, CIMUS, University of Santiago de Compostela-Instituto de Investigación Sanitaria, 15782 Santiago de Compostela, Spain; 12 Metabolic Research Laboratory, Clínica Universidad de Navarra, University of Navarra-IdiSNA, 31008 Pamplona, Spain; 13 Eating Disorders Program, Department of Psychiatry, University of California, San Francisco, CA 94143, USA; Daniel.LeGrange@ucsf.edu; 14 Section of Eating Disorders, Department of Psychological Medicine, Institute of Psychiatry, Psychology and Neuroscience, King’s College London, London WC2R 2LS, UK; kate.tchanturia@kcl.ac.uk (K.T.); katie.rowlands@kcl.ac.uk (K.R.); janet.treasure@kcl.ac.uk (J.T.); 15 Eating Disorders Unit, Department of Child and Adolescent Psychiatry, Medical University of Vienna, 1090 Vienna, Austria; Andreas.karwautz@meduniwien.ac.at (A.K.); michael.zeiler@meduniwien.ac.at (M.Z.); 16 Parkland Klinik, 34537 Bad Wildungen, Germany; Hartmut.imgart@parkland-klinik.de (H.I.); Annika.zanko@parkland-klinik.de (A.Z.); 17 Department of Neuroscience, University of Padua and Neuroscience Center (PNC), 35122 Padua, Italy; angela.favaro@unipd.it; 18 Clinical Psychology, Faculty of Psychology and Educational Sciences, KU Leuven, 3000 Leuven, Belgium; laurence.claes@kuleuven.be; 19 Clinical Psychology, Faculty of Medicine and Health Sciences, University Antwerp, 2000 Antwerp, Belgium; 20 D. Uznadze Institute of Psychology, Ilia State University, 0162 Tbilisi, Georgia; ia.shekriladze@iliauni.edu.ge; 21 Child and Adolescent Psychiatry and Psychology Department, Hospital Sant Joan de Déu- Institut de Recerca Sant Joan de Déu, 08950 Esplugues de Llobregat, Spain; eduardo.serrano@sjd.es (E.S.-T.); raquel.cecilia@sjd.es (R.C.-C.); 22 Department of Psychiatry, Germans Trias i Pujol University Hospital-IGTP, 08916 Barcelona, Spain; teresa.rangil@uab.cat (T.R.); mloran.germanstrias@gencat.cat (M.E.L.-M.); 23 Department of Psychiatry and Legal Medicine, Universitat Autònoma de Barcelona, 08193 Barcelona, Spain; 24 Department of Psychiatry, Hospital de la Santa Creu i Sant Pau-Institut d’Investigació Biomèdica Sant Pau (IIB-Sant Pau)-Universitat Autònoma de Barcelona (UAB), 08041 Barcelona, Spain; jsoriano@santpau.cat (J.S.-P.); mar.carceller@e-campus.uab.cat (M.C.-S.); 25 Centro de Investigación Biomédica en Red de Salud Mental (CIBERSAM), ISCIII, 28029 Madrid, Spain; mtplana@clinic.cat (M.T.P.); montserrat.graell@salud.madrid.org (M.G.); andres.gomez@scsalud.es (J.A.G.-d.B.); 26 Centro de Diagnóstico de Enfermedades Moleculares, Universidad Autónoma de Madrid, 28049 Madrid, Spain; rnavarrete@cbm.csic.es; 27 FITA Foundation, 08006 Barcelona, Spain; mlozano@fitafundacion.org (M.L.); rlinares@fitafundacion.org (R.L.); 28 Infectious Diseases Department, Hospital Universitari Bellvitge-Institut de Investigació Biomedica de Bellvitge (IDIBELL)-Institut Català d’Oncologia-Hospitalet, 08908 Barcelona, Spain; cgudiol@bellvitgehospital.cat (C.G.); jcarratala@bellvitgehospital.cat (J.C.); 29 REIPI (Spanish Network for Research in Infectious Disease), Instituto de Salud Carlos III, 28029 Madrid, Spain; 30 Department of Clinical Sciences, School of Medicine and Health Sciences, University of Barcelona, 08907 Barcelona, Spain; 31 CIBER de Enfermedades Infecciosas (CIBERINFEC), Instituto de Salud Carlos III, 28029 Madrid, Spain; 32 Institute of Neuroscience, Hospital Clínic de Barcelona, 08036 Barcelona, Spain; 33 Department of Child and Adolescent Psychiatry and Psychology, 2017SGR881, Institute of Neurosciences, Hospital Clinic de Barcelona, 08036 Barcelona, Spain; 34 Child and Adolescent Psychiatry and Psychology Service, Child Hospital Niño Jesus, 28009 Madrid, Spain; 35 Psychiatry Service, University of Salamanca Healthcare Complex (USHC)-Institute of Biomedicine of Salamanca (IBSAL)-University of Salamanca, 37008 Salamanca, Spain; dgonzalezp@saludcastillayleon.es; 36 Unidad de Trastornos de la Conducta Alimentaria, Hospital Universitario “Marqués de Valdecilla”, Avda, Valdecilla s/n, 39011 Santander, Spain; 37 Instituto de Investigación Valdecilla (IDIVAL), 39011 Santander, Spain; 38 Facultad de Psicología, Universidad Autónoma de Madrid, 28049 Madrid, Spain; anarosa.sepulveda@uam.es; 39 Psychotherapy and Psychopathology Research Unit—Psychology Research Center, School of Psychology, University of Minho, 4710-057 Braga, Portugal; pmachado@psi.uminho.pt; 40 Department of Clinical Sciences Lund, Psychiatry, Faculty of Medicine, Lund University, 221 00 Lund, Sweden; anders_c.hakansson@med.lu.se; 41 Gambling Disorder Unit, Malmö Addiction Center, 205 02 Malmö, Sweden; 42 Institute of Behavioral Sciences, Semmelweis University, 1085 Budapest, Hungary; turyferenc@gmail.com; 43 1st Department of Paediatrics, Semmelweis University, 1085 Budapest, Hungary; paszthy@gyer1.sote.hu; 44 Safra Children’s Hospital, Chaim Sheba Medical Center, Tel Hashomer 52 621, Israel; Daniel.Stein@sheba.health.gov.il; 45 Department of Psychiatry, 1st Medical Faculty of Charles University, 11000 Prague, Czech Republic; hana.papezova@lf1.cuni.cz (H.P.); jana.gricova@lf1.cuni.cz (J.G.); 46 Eating Disorders Center, Vilnius University Vilnius, 01513 Vilnius, Lithuania; bax.brigita@gmail.com; 47 Institute of Physiology of Komi Science Centre of the Ural Branch of the Russian Academy of Sciences, 167982 Syktyvkar, Russia; borisenkov@physiol.komisc.ru (M.F.B.); s.v.popov@inbox.ru (S.V.P.); 48 Laboratory for Chronobiology and Chronomedicine, Department of Biology, Tyumen State Medical University, 625023 Tyumen, Russia; gubin@tyumsmu.ru; 49 Tyumen Cardiology Research Institute, Tomsk Research Medical Center, 634009 Tyumen, Russia; 50 Department of Biological & Medical Physics UNESCO, Tyumen State Medical University, 625023 Tyumen, Russia; petrov@tyumsmu.ru; 51 Department of Therapy and Endocrinology, Tyumen State Medical University, 625023 Tyumen, Russia; isakovadn@tyumsmu.ru; 52 Institute of Internal and Preventive Medicine–Branch of the Institute of Cytology and Genetics, Siberian Branch of Russian Academy of Sciences, 630090 Novosibirsk, Russia; svetlana3548@gmail.com; 53 Department of Psychiatry, Seoul Paik Hospital-Inje University, Seoul 01757, Korea; youlri.kim@gmail.com; 54 Department of Psychiatry, School of Medicine, International University of Health and Welfare, Narita 286-8686, Japan; michiko.nakazato@nifty.ne.jp; 55 CESP, Université Paris-Saclay, UVSQ, INSERM U 1178, 94805 Villejuif, France; nathalie.godart@fsef.net; 56 Department of Psychiatry, Institut Mutualiste Montsouris, School of Medicine, Université Paris Descartes, 75006 Paris, France; 57 UFR des Sciences de la Santé Simone Veil (UVSQ), Praticienne Hospitalière, Fondation Santé des Etudiants de France, 78180 Paris, France; 58 Department of Political Science, Vytautas Magnus University, 44248 Kaunas, Lithuania; rvvoren@gmail.com; 59 Institute of Psychiatry of Taras Shevchenko, National University of Kyiv, 01033 Kyiv, Ukraine; tatiana.ilnitskaya.14@gmail.com; 60 Department of Clinical Psychology, Shanghai Mental Health Center, Shanghai Jiao Tong University School of Medicine, 280, Shanghai 200030, China; chenjue2088@163.com; 61 Schön Klinik Roseneck, 83209 Prien am Chiemsee, Germany; UVoderholzer@schoen-klinik.de; 62 Dipartimento di Salute Mentale e Fisica e Medicina Preventiva, Universitá degli Studi della Campania “Luigi Vanvitelli”, 80138 Naples, Italy; alessiomaria.monteleone@unicampania.it

**Keywords:** eating disorders, COVID-19 lockdown, COVID-19 Isolation Eating Scale (CIES), eating symptoms, psychological impact

## Abstract

Background. The COVID-19 lockdown has had a significant impact on mental health. Patients with eating disorders (ED) have been particularly vulnerable. Aims. (1) To explore changes in eating-related symptoms and general psychopathology during lockdown in patients with an ED from various European and Asian countries; and (2) to assess differences related to diagnostic ED subtypes, age, and geography. Methods. The sample comprised 829 participants, diagnosed with an ED according to DSM-5 criteria from specialized ED units in Europe and Asia. Participants were assessed using the COVID-19 Isolation Scale (CIES). Results. Patients with binge eating disorder (BED) experienced the highest impact on weight and ED symptoms in comparison with other ED subtypes during lockdown, whereas individuals with other specified feeding and eating disorders (OFSED) had greater deterioration in general psychological functioning than subjects with other ED subtypes. Finally, Asian and younger individuals appeared to be more resilient. Conclusions. The psychopathological changes in ED patients during the COVID-19 lockdown varied by cultural context and individual variation in age and ED diagnosis. Clinical services may need to target preventive measures and adapt therapeutic approaches for the most vulnerable patients.

## 1. Introduction

The COVID-19 pandemic has been a challenge for governments and health care professionals. The lockdown has been a worldwide response to control the spread of the disease. Although the measures taken have been effective in reducing the transmission of the infection, health professionals have expressed concerns about the mental health consequences that can result from social isolation and restrictions to daily life [1,2,3].

The psychological impact of lockdown in history and in the current context has been considered [4]. Higher levels of negative emotions such as anxiety, depression, anger, guilt or even posttraumatic stress symptoms have been reported [4,5]. A more profound impact has been observed in individuals with chronic diseases and mental illness [1,6,7]. Patients with eating disorders (ED) have been found to be at risk of adverse psychological consequences in the context of the COVID-19 pandemic [8,9].

Several studies have highlighted the emotional distress due to lockdown in patients with an ED, reporting high levels of anxiety, depression, and post-stress traumatic symptoms that may persist after lockdown [10,11,12]. Social distancing might obstruct adaptive strategies to deal with psychological distress [3,9,13] and maladaptive coping strategies, such as engaging in substance abuse and potentially addictive behaviors (e.g., gaming), may be adopted [14,15].

Changes in eating behaviors, exercise and weight/body mass index (BMI) have been detected both in the general population and in patients with an ED [16,17]. Emotional disturbances secondary to environmental changes and “food insecurity” have been considered as some possible explanatory factors [17,18,19]. Reduced social support, low self-direction, childhood trauma, and insecure attachment or difficulties in emotion regulation are vulnerability factors leading to psychological distress in lockdown, which can be associated with disturbed eating patterns [11,20,21,22,23].

Increased dietary restriction and physical activity in patients with anorexia nervosa (AN) or higher frequency of binge episodes among patients with bulimia nervosa (BN) and binge eating disorder (BED) have been reported during lockdown [10,11,21,24,25]. However, there are few studies focusing on the evolution of ED symptoms after lockdown and these have yielded mixed results [10,11]. A differential impact on eating and general psychopathology has been assessed in patients with an ED when compared with the general population [11,16]. This may vary with the ED subtype [26]. Moreover, age may be a possible factor to consider when evaluating clinical changes in the context of lockdown. In this line, younger age [1,7], together with cultural and socio-demographic factors may modulate them [27,28].

In order to assess the global effect of lockdown due to the COVID-19 pandemic in patients with a current diagnosis of ED, an international group of clinical and research experts developed the COVID Isolation Eating Scale (CIES), which has been translated in nineteen languages [26]. The study by Fernandez-Aranda, Munguía et al. [26] provided evidence of the psychometric robustness of the Spanish version of the CIES, with an adequate goodness-of-fit for the confirmatory factor analysis and good to excellent Cronbach alpha values. Preliminary data suggested that the effects of lockdown differed between ED subtypes, whereby patients with other specified feeding and eating disorders (OSFED) reported the highest global impairment [26].

To the best of our knowledge, this is the first observational study to analyze clinical changes in patients with ED longitudinally during lockdown, from a multicenter and international perspective. Both child/adolescent and adult populations were assessed using the CIES. The aims of the present study were: (1) to explore eating symptoms and behavioral changes, as well as other psychopathological features in the context of lockdown, and (2) to examine whether ED subtypes, age and geography moderated this effect.

## 2. Materials and Methods

### 2.1. Participants

The sample comprised *N* = 829 participants, from European (Spain, *n* = 300; Austria, *n* = 43; Germany, *n* = 103; Russia, *n* = 119; Portugal, *n* = 28; Lithuania, *n* = 23; Czech Republic, *n* = 50; Ukraine, *n* = 10) and Asian (China, *n* = 92; Korea, *n* = 50; Japan, *n* = 11) private and public ED units. Participants were diagnosed according to DSM-5 criteria [29] by expert clinical psychologists and psychiatrists using a semi-structured clinical interview (SCID-5) [30].

### 2.2. Assessment

The COVID Isolation Eating Scale (CIES) [26] is a self-report questionnaire that assesses the impact of lockdown on patients with ED and/or obesity and has been translated in 19 languages [26]. Supplementary Material contains the English version of the scale. It is composed of 4 subscales:

I. Circumstances of the lockdown (eight items).
II. Effects of the lockdown on eating symptoms (thirteen items): it evaluates symptomatology of AN, BN, BED and OSFED, according with the DSM-5. Comorbidity with other psychiatric disorders or diabetes is assessed.
III. Reaction to the lockdown (34 items): it evaluates the effects of the confinement on eating behaviors, attitudes and habits, anxious-depressive symptoms, emotion dysregulation, and other symptomatology associated with substance use disorders and behavioral addictions.
IV. The evaluation of remote interventions (thirteen items) assesses acceptance, general satisfaction, and motivation for virtual interventions.

The last three subscales are answered in a five-point Likert scale. Section 2 and Section 3 consider two moments of time, before confinement and the current moment of time.

According to the factorial analysis [26], five factors were identified. Factor 1 was defined by the items measuring eating related symptoms; Factor 2 by the items measuring the effects of lockdown on the eating-related style; Factor 3 by the items assessing anxiety and depressive symptoms; Factor 4 was defined by the items related to emotion regulation; and Factor 5 by those that evaluate telemedicine.

Only the subscales I, II and III (that correspond to the factor 1, 2, 3 and 4) were used in this study. Therefore, no results referring to the acceptance of virtual are reported (subscale 4, factor 5).

### 2.3. Additional Assessment

Socio-demographic and clinical information (i.e., age, ED subtype, and variables related to COVID-19 and lockdown) were also obtained through the CIES questionnaire.

### 2.4. Procedure

All the participants were already involved in outpatient ED treatment in specialized units of the different countries. The subjects were asked by therapists from each center to voluntarily complete the study questionnaire (i.e., CIES), translated into participants’ languages [26]. Data collection took place retrospectively between August 2020 and January 2021.

The study was approved by the Clinical Research Ethics Committee of the leading University Hospital (Bellvitge University Hospital) (PR239/20), and informed written consent was obtained from all participants.

### 2.5. Statistical Analysis

Statistical analysis was performed with Stata17 for Windows [31]. The assessment of the pre-post changes in the quantitative measures was carried out through repeated measures analysis of variance (repeated-ANOVA), while the McNemar test was used for paired nominal data.

Additionally, the differences post-pre values were generated for the scores registered in the CIES, and for the weight (kg) and the BMI (kg/m^2^) (values equal to zero in these new variables indicate absence of change; negative values indicate a decreasing pre-post change; and positive values indicate an increasing pre-post change). Next, ANOVA procedures compared the mean differences between the diagnostic subtypes, the groups of age, and the continent. 

In this study, the statistical analyses were adjusted by the sociodemographic sex and age, due to the differences between the groups (defined by the diagnostic subtypes and the origin of the samples).The effect size was estimated with Cohen’s-h coefficient for the differences between the proportions and with Cohen’s-d for the differences between the means (null effect size was considered for |h| < 0.20 or |d| < 0.20, low-poor for |h| > 0.20 or |d| > 0.20, moderate-medium for |h| > 0.50 or |d| > 0.50 and large-high for |h| > 0.80 or |d| > 0.80) [32,33]. Finner’s method controlled the increase in the Type-I error due the use of multiple significance tests [34].

## 3. Results

### 3.1. Characteristics of the Participants

Table S1 (Supplementary Material) displays sociodemographic and clinical characteristic of the sample during the lockdown, as well as the comparison between the diagnostic types, the age groups, and the different continents. For the total sample, the mean age was 27.9 years old (*SD* = 12.3). Most participants were women (70.4%), lived with other people during the lockdown (78.9%), did not have to take care of others (73.8%), were not infected with COVID-19 (92.8%), did not know anyone close to them who was infected with COVID-19 (84.8%), were working (53.8%), and reported a minimal financial loss (73.5%).

### 3.2. Comparison between Diagnostic Subtypes of Changes Pre to Post Lockdown

Table 1 contains the changes in the CIES subscales scores between the pre- and post-lockdown within each diagnostic subtype. After adjusting by sex and age, patients with AN reported a significant worsening in eating style and alcohol use. Patients with BN had an increase in weight, and a decrease in ED symptoms, but emotion dysregulation and alcohol use were increased. Individuals with BED reported increased weight, an impaired eating style and had an increase in anxiety-depression symptoms. The group with OSFED reported an increase in anxiety-depression symptoms and emotion dysregulation.

**Figure 1:**
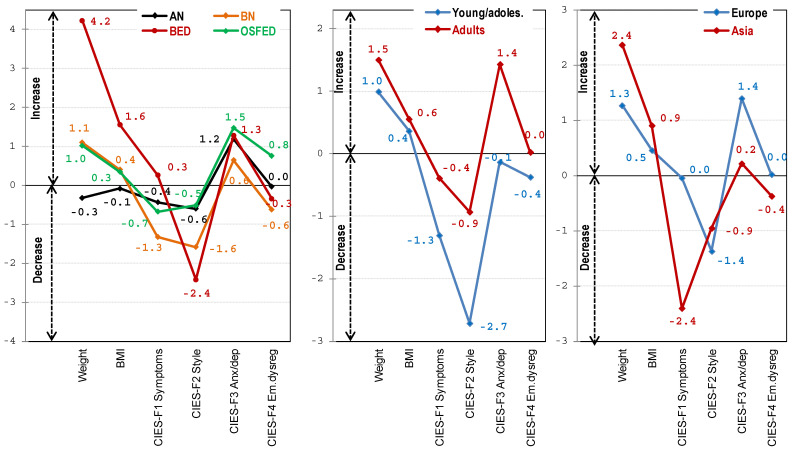
Differences (post-pre changes) for the weight, the BMI and the CIES factors.

Table 2 shows the comparison between the diagnostic ED subtypes for post-pre changes (defined as the difference between the measures at the end of the follow-up and the post lockdown versus the pre lockdown). The first line-plot displayed in Figure 1 also contains the mean changes within each measure (mean values close to the horizontal line at value 0 represent absence of pre-post changes). After adjusting by sex and age, compared with other groups, patients with BED were characterized by the highest increase in weight, BMI, and the CIES-F1 ED symptoms. For the CIES-F2 eating style, BED and BN achieved statistically equal changes, which represented a higher decrease compared with AN, and OSFED. For CIES-F3 anxiety-depression and CIES-F4 emotional dysregulation, OSFED registered the highest increase compared with the other diagnostic conditions. 

### 3.3. Comparison between the Groups of Age for the Changes during the Lockdown

Table 3 contains the results of the repeated-ANOVA assessing the changes between the pre- and post-lockdown within each age group (young/adolescents versus adults). These analyses were adjusted by the ED subtype and sex. Among young/adolescents, a significant decrease was observed for the CIES-F2 eating style. Adult patients reported increased weight, BMI and CIES-F3 anxiety-depression symptoms, decreased CIES-F2 eating style, and the likelihood of alcohol and other illegal drug use.

The results of the ANOVA procedures comparing between the age group the differences generated between the values measured at post- and pre-lockdown are shown in Table 4 (ANOVA procedures adjusted by the ED subtype and sex) (see also the second line-plot of Figure 1). Statistical differences were achieved by comparing the mean changes in the CIES-F2 eating style and the CIES-F3 anxiety-depression symptoms. 

### 3.4. Comparison between Continents for the Changes during the Lockdown

Table 5 contains the results of the repeated-ANOVA (adjusted by the ED subtype, sex, and age) assessing the changes between the pre- and post-lockdown in the weight, the BMI and the CIES scores. Separated/stratified analyses have been performed within each continent (Europe and Asia). Among the European patients, a significant increase was observed in weight, BMI and the CIES-F3 anxiety-depression symptoms. Besides, a significant decrease in the CIES-F2 eating style and in the likelihood of alcohol and other illegal drug use was reported. In Asian patients, while an increase in weight and BMI was obtained, a decrease in the CIES-F1 ED symptoms was found. 

Finally, the results of the ANOVA comparing the differences post- and pre-lockdown between the geographical area are shown in Table 6 (results adjusted by the ED subtype, sex, and age) (see also the third line-plot of Figure 1). Statistical differences were achieved comparing the mean changes in the CIES-F1 ED symptoms (higher decrease for Asian patients). 

## 4. Discussion

The aim of this study was to compare the psychopathological effects in the context of lockdown due to the COVID-19 pandemic in patients with ED from different continents (i.e., Europe and Asia). Secondly, we examined if these differences pre/post lockdown varied by ED subtype, age, and continental provenance.

### 4.1. ED Subtypes and Changes during Lockdown

In line with previous studies [26] the findings highlight a differential psychopathological impact during lockdown according to the ED diagnosis. Patients diagnosed with BN and BED reported weight gain after lockdown, the BED group experiencing the greatest weight changes when comparing by ED subtypes. It is possible that a sedentary lifestyle favored by “stay-at-home” measures and mobility restrictions during lockdown [35] may play a pivotal role in these weight changes [36,37]. Some of the increased eating behaviors related to “food insecurity” or boredom, such as snacking [35], could also lead to weight gain [16,17]. In this line, individuals with BED and BN showed a higher impairment in their eating style in comparison with the other ED subtypes. Moreover, previous studies have recognized an increased vulnerability to weight gain in individuals with overweight and obesity [38], conditions that are usually presented in individuals with BN and BED [39,40].

Eating behaviors have been described as maladaptive strategies to cope with emotional distress [13,18,22]. However, ED symptoms and emotion dysregulation were reduced in BN, which aligns with other findings [10,41]. The continued presence of other people at home and maintaining daily routines [4] are two significant socio-demographic features that characterized our sample, and may have allowed patients with BN to reduce binge episodes and purging. Moreover, the observed decrease in other maladaptive behaviors after lockdown (i.e., alcohol consumption) has been previously described in an international non-clinical study [35].

Individuals with BED reported the highest impact on ED symptoms when comparing ED subtype groups, although no significant changes in ED symptoms pre- and post-lockdown were individually reported in this group. A greater illness perception among these patients together with a higher motivation for change [42], and the weight gain during lockdown, could encourage them to improve their eating style (e.g., non abuse of certain palatable foods).

Changes in eating style have also been described in patients with AN, which may suggest an increased control over food intake during lockdown. Changes in diet habits have been observed both in general and clinical populations during lockdown [16], the restricting pattern being commonly mentioned [24]. Surprisingly, and in contrast to other studies [12,43], they did not report significant modification in weight/BMI, ED symptoms nor other psychological features. Furthermore, a reduction in alcohol use was described in this ED subtype after lockdown.

Regarding OSFED group, both psychopathology and emotion regulation worsened during lockdown. This aligns with previous research which found that individuals with OSFED deteriorated in adverse situations [44,45]. Not only they did experience a greater psychological impairment than subjects from other ED groups but this impairment also persisted after lockdown [10,11,23].

### 4.2. Age Differences Regarding Changes during Lockdown

Overall young/adolescent patients had a significant improvement in eating habits in comparison to older individuals. The adult group reported significant weight/BMI changes and higher psychological impact than the former one. These results differed from those described in the general population in which younger people had a poorer adjustment to lockdown [1,7]. One possible explanation for our results could be that older age is linked to patients with BED, who in our study reported a greater psychopathological impact than other ED subtypes. Besides, younger people may be supervised and accompanied more frequently by others, constituting a possible protective factor [4]. Furthermore, young people are usually more familiar with the use of social media, which might imply a better adaptation to the online modality, promoting therapeutic adherence and the maintenance of academic/work routines, as well as social contact [4,13].

### 4.3. Influence of Continental Provenance on the Changes during Lockdown

Both European and Asian patients experienced weight gain during lockdown, as has been described in clinical and general populations [36,37,46], without significant differences between groups. In comparison with European patients, participants from Asia reported an improvement of their ED symptoms. These findings were not in line with other studies exploring the impact of lockdown on the Asian population [46]. Individual differences in personality and coping features have been reported between countries [27,28] which might lead to a differential adjustment to stress such as the COVID-19 pandemic [47].

### 4.4. Strengths and Limitations

The large sample size with international participation using a validated and homogeneous method of evaluation is a strength of this study. Moreover, the analysis related to age and provenance were adjusted for the ED subtype. However, the observational design of the study and the voluntary nature of participation in it could be some of the limitations of the present work. Furthermore, the presence of memory bias due to the retrospective nature of the assessment and the heterogeneity of the sample must be mentioned. Even though initial evidence regarding clinical changes in ED patients during lockdown has been measured by the CIES, future studies should consider involving other ED severity questionnaires.

## 5. Conclusions

In this international study of the adjustment of patients with an ED during lockdown, we observed differences that varied according to ED subtype, age, and provenance. Individuals with BED showed higher worsening of eating symptoms and change in weight during lockdown than those from other ED subtypes. The greatest psychological impairment was described in the OSFED group. Finally, young and Asian patients appeared to be more resilient. These findings may enhance preventive and therapeutic approaches in similar future circumstances.

## Figures and Tables

**Table 1:** Assessment of the post-pre changes stratified by ED subtype.

	Pre		Post			
**Anorexia (*n* = 370)**	Mean	SD	Mean	SD	*p*	|d|
Weight (kg)	48.29	9.02	48.27	8.23	0.954	0.00
BMI (kg/m^2^)	17.92	3.06	17.94	3.00	0.862	0.01
CIES-F1 ED symptoms	13.94	6.93	13.32	7.46	0.089	0.09
CIES-F2 Eating style	10.96	8.75	9.98	8.71	0.005 *	0.11
CIES-F3 Anxiety-depression	17.20	9.62	17.93	9.85	0.089	0.07
CIES-F4 Emotion dysregulation	8.51	5.09	8.27	5.26	0.247	0.05
	*n*	*%*	*n*	*%*	*p*	*|h|*
Tobacco	52	14.1%	52	14.1%	1.00	0.00
Alcohol	57	15.4%	38	10.3%	0.001 *	0.15
Other illegal drugs	33	8.9%	26	7.0%	0.143	0.07
Behavioral addictions	244	65.9%	242	65.4%	0.875	0.01
**Bulimia (*n* = 148)**	Mean	SD	Mean	SD	*p*	|d|
Weight (kg)	61.09	14.52	62.26	13.89	0.026 *	0.08
BMI (kg/m^2^)	22.27	5.07	22.70	4.84	0.025 *	0.09
CIES-F1 ED symptoms	19.78	6.82	18.39	7.16	0.048 *	0.20
CIES-F2 Eating style	21.96	10.15	20.28	10.79	0.073	0.16
CIES-F3 Anxiety-depression	20.36	9.02	20.84	9.61	0.467	0.05
CIES-F4 Emotion dysregulation	9.91	4.83	9.22	5.28	0.045 *	0.14
	*n*	*%*	*n*	*%*	*p*	|h|
Tobacco	42	28.4%	46	31.1%	0.388	0.06
Alcohol	62	41.9%	45	30.4%	<0.001 *	0.24
Other illegal drugs	30	20.3%	22	14.9%	0.115	0.14
Behavioral addictions	108	73.0%	106	71.6%	0.839	0.03
**BED (*n* = 113)**	Mean	SD	Mean	SD	*p*	|d|
Weight (kg)	95.27	33.79	99.18	31.49	<0.001 *	0.12
BMI (kg/m^2^)	33.63	10.38	35.08	9.63	<0.001 *	0.14
CIES-F1 ED symptoms	14.10	7.26	14.57	6.72	0.449	0.07
CIES-F2 Eating style	20.88	11.30	18.86	11.60	0.010 *	0.18
CIES-F3 Anxiety-depression	17.66	10.37	19.45	11.37	0.004 *	0.16
CIES-F4 Emotion dysregulation	8.99	5.91	8.88	5.87	0.745	0.02
	*n*	*%*	*n*	*%*	*p*	|d|
Tobacco	29	25.7%	28	24.8%	1.00	0.02
Alcohol	36	31.9%	31	27.4%	0.405	0.10
Other illegal drugs	18	15.9%	13	11.5%	0.332	0.13
Behavioral addictions	73	64.6%	79	69.9%	0.263	0.11
**OSFED (*n* = 198)**	Mean	SD	Mean	SD	*p*	|d|
Weight (kg)	81.68	25.86	82.25	26.29	0.280	0.02
BMI (kg/m^2^)	28.58	8.06	28.77	8.13	0.340	0.02
CIES-F1 ED symptoms	13.74	5.95	13.35	5.70	0.266	0.07
CIES-F2 Eating style	13.94	9.14	13.99	9.12	0.924	0.00
CIES-F3 Anxiety-depression	13.99	10.03	16.17	10.90	<0.001 *	0.21
CIES-F4 Emotion dysregulation	6.73	5.08	7.80	5.86	<0.001 *	0.20
	*n*	*%*	*n*	*%*	*p*	|h|
Tobacco	58	29.3%	65	32.8%	0.092	0.08
Alcohol	57	28.8%	51	25.8%	0.286	0.07
Other illegal drugs	13	6.6%	16	8.1%	0.629	0.06
Behavioral addictions	123	62.1%	131	66.2%	0.152	0.08

BMI: body mass index. BED: binge eating disorder. OSFED: other specified feeding eating disorders. SD: standard deviation. * Bold: significant comparison. Results adjusted by sex and age.

**Table 2:** Comparison of the post-pre differences by the ED subtype.

	Anorexia (AN)		Bulimia (BN)		BED		OSFED		Significant
	*n* = 370		*n* = 148		*n* = 113		*n* = 198		Pairwise
	Mean	SD	Mean	SD	Mean	SD	Mean	SD	Comparisons
Weight (kg)	−0.32	6.66	1.10	6.32	4.22	11.58	1.02	7.43	BED ≠ (AN = BN = OSFED)
BMI (kg/m^2^)	−0.09	2.54	0.40	2.31	1.55	4.13	0.35	2.71	BED ≠ (AN = BN = OSFED)
CIES-F1 ED symptoms	−0.43	7.03	−1.32	8.52	0.26	6.54	−0.67	4.90	BED ≠ (AN = BN = OSFED)
CIES-F2 Eating style	−0.60	6.72	−1.58	11.34	−2.42	8.12	−0.52	6.69	(BED = BN) ≠ (AN = OSFED)
CIES-F3 Anxiety-dep.	1.19	8.28	0.65	8.28	1.28	6.53	1.47	7.04	OSFED ≠ (AN = BN = BED)
CIES-F4 Emot.dysreg.	−0.03	4.06	−0.62	4.25	−0.34	3.73	0.75	3.94	OSFED ≠ (AN = BN = BED)

BMI: body mass index. BED: binge eating disorder. OSFED: other specified feeding eating disorders. SD: standard deviation.

**Table 3:** Assessment of the post-pre changes stratified by groups of age.

	Pre		Post			
**Age: young/adolescents (*n* = 172)**	Mean	SD	Mean	SD	*p*	|d|
Weight (kg)	63.49	13.52	65.44	13.51	0.073	0.14
BMI (kg/m^2^)	22.74	4.00	23.37	3.97	0.119	0.16
CIES-F1 ED symptoms	14.83	6.73	13.32	7.52	0.213	0.21
CIES-F2 Eating style	16.54	9.23	12.75	8.60	0.003 *	0.43
CIES-F3 Anxiety-depression symptoms	15.96	8.89	15.46	8.83	0.701	0.06
CIES-F4 Emotion dysregulation	8.84	5.24	8.39	5.33	0.537	0.09
	*n*	*%*	*n*	*%*	*p*	*|h|*
Tobacco	14	9.6%	16	11.1%	0.566	0.05
Alcohol	18	9.7%	9	3.1%	0.118	0.28
Other illegal drugs	9	6.0%	8	5.8%	0.945	0.01
Behavioral addictions	125	79.7%	127	80.1%	0.948	0.01
**Age: adults (*n* = 657)**	Mean	SD	Mean	SD	*p*	|d|
Weight (kg)	72.18	27.82	73.62	27.96	<0.001 *	0.05
BMI (kg/m^2^)	25.79	9.00	26.32	9.10	<0.001 *	0.06
CIES-F1 ED symptoms	15.54	7.14	15.22	6.96	0.237	0.04
CIES-F2 Eating style	17.17	10.60	16.32	10.52	0.008 *	0.08
CIES-F3 Anxiety-depression symptoms	17.69	10.13	19.26	10.57	<0.001 *	0.15
CIES-F4 Emotion dysregulation	8.61	5.27	8.70	5.53	0.546	0.02
	*n*	*%*	*n*	*%*	*p*	|h|
Tobacco	167	26.2%	175	27.4%	0.235	0.03
Alcohol	194	31.4%	156	25.4%	<0.001 *	0.13
Other illegal drugs	85	13.9%	69	11.0%	0.025 *	0.09
Behavioral addictions	423	65.0%	431	66.6%	0.272	0.03

BMI: body mass index. SD: standard deviation. * Bold: significant comparison. Results adjusted by ED subtype and sex.

**Table 4:** Comparison of the post-pre differences by the groups of age.

	Young/Adolescents		Adults			
	*n* = 172		*n* = 657			
	Mean	SD	Mean	SD	*p*	|d|
Weight (kg)	0.99	7.16	1.50	7.86	0.480	0.07
BMI (kg/m^2^)	0.36	2.66	0.56	2.89	0.471	0.04
CIES-F1 ED symptoms	−1.30	7.94	−0.39	6.50	0.154	0.13
CIES-F2 Eating style	−2.71	8.17	−0.93	7.85	0.017 *	0.22
CIES-F3 Anxiety-depression symptoms	−0.13	8.55	1.43	7.52	0.030 *	0.19
CIES-F4 Emotion dysregulation	−0.37	4.77	0.03	3.84	0.289	0.09

BMI: body mass index. SD: standard deviation. * Bold: significant comparison. Results adjusted by ED subtype and sex.

**Table 5:** Assessment of the post-pre changes stratified by continent.

	Pre		Post			
**Europe (*n* = 676)**	Mean	SD	Mean	SD	*p*	|d|
Weight (kg)	72.99	27.78	74.20	28.24	0.001 *	0.04
BMI (kg/m^2^)	26.06	8.95	26.49	9.14	0.001 *	0.05
CIES-F1 ED symptoms	14.94	6.61	15.09	7.06	0.587	0.02
CIES-F2 Eating style	16.29	9.73	15.03	9.82	<0.001 *	0.13
CIES-F3 Anxiety-depression symptoms	17.00	9.65	18.51	10.34	<0.001 *	0.15
CIES-F4 Emotion dysregulation	8.54	5.13	8.58	5.46	0.805	0.01
	*n*	*%*	*n*	*%*	*p*	|h|
Tobacco	166	28.2%	173	29.3%	0.306	0.02
Alcohol	172	30.8%	132	22.9%	<0.001 *	0.18
Other illegal drugs	75	14.5%	58	10.2%	0.003 *	0.13
Behavioral addictions	440	66.9%	452	68.4%	0.209	0.03
**Asia (*n*=153)**	Mean	SD	Mean	SD	*p*	|d|
Weight (kg)	56.19	15.41	58.01	14.99	0.038 *	0.12
BMI (kg/m^2^)	20.79	4.95	21.51	4.91	0.030 *	0.15
CIES-F1 ED symptoms	17.91	8.14	15.55	7.53	0.006 *	0.30
CIES-F2 Eating style	19.78	12.44	18.61	12.15	0.314	0.10
CIES-F3 Anxiety-depression symptoms	19.02	10.74	19.13	10.59	0.899	0.01
CIES-F4 Emotion dysregulation	9.13	5.75	8.61	5.73	0.241	0.09
	*n*	*%*	*n*	*%*	*p*	*|h|*
Tobacco	15	12.0%	18	14.6%	0.167	0.08
Alcohol	40	28.6%	33	25.6%	0.354	0.07
Other illegal drugs	19	13.6%	19	14.4%	0.736	0.02
Behavioral addictions	108	68.0%	106	73.0%	0.284	0.11

BMI: body mass index. SD: standard deviation. * Bold: significant comparison. Results adjusted by ED subtype, sex, and age.

**Table 6:** Comparison of the post-pre differences by continent.

	Europe		Asia			
	*n* = 676		*n* = 153			
	Mean	SD	Mean	SD	*p*	|d|
Weight (kg)	1.27	7.47	2.37	8.73	0.138	0.13
BMI (kg/m^2^)	0.46	2.73	0.91	3.28	0.100	0.15
CIES-F1 ED symptoms	−0.04	6.32	−2.40	8.43	<0.001 *	0.32
CIES-F2 Eating style	−1.37	6.90	−0.95	11.48	0.583	0.04
CIES-F3 Anxiety-depression symptoms	1.40	7.54	0.22	8.62	0.113	0.15
CIES-F4 Emotion dysregulation	0.02	3.94	−0.37	4.47	0.309	0.09

BMI: body mass index. SD: standard deviation. * Bold: significant comparison. Results adjusted by ED subtype, sex, and age.

## Data Availability

Individuals may inquire with Fernández-Aranda regarding availability of the data as there is ongoing studies using the data. To avoid overlapping research efforts, Fernández-Aranda will consider a request on a case-by-case basis.

## Supplementary Materials

The following supporting information can be downloaded at: https://www.mdpi.com/article/10.3390/nu14010100/s1, Table S1. Descriptive for the age, sex, and the confinement context/ Covid Isolation Eating Scale (CIES)-English version.
