# Supplementary material for: Impact of COVID-19 Lockdown in Eating Disorders: A Multicentre Collaborative International Study
Source: Nutrients. 2021 Dec 27;14(1):100. doi: 10.3390/nu14010100 (PMC8746935; doi:10.3390/nu14010100)
Supplement: Supplementary file 1 [file nutrients-14-00100-s001.zip › nutrients-1463540-supplementary.pdf]

SUPPLEMENTARY MATERIAL

Table S1 Descriptive for the age, sex and the confinement context

		Total *N=829*		Anorexia *N=370*		Bulimia *N=148*		BED *N=113*		OSFED *N=198*		*p*
Age (yrs-old); *mean - SD*		27.90	12.32	21.88	8.61	26.76	9.03	33.84	12.02	36.59	13.83	0.001*
Sex; *n* - %	Female	584	70.4%	299	80.8%	111	75.0%	66	58.4%	108	54.5%	0.001*
	Male	245	29.6%	71	19.2%	37	25.0%	47	41.6%	90	45.5%	
Contextual lock-down		*n*	%	*n*	%	*n*	%	*n*	%	*n*	%	*p*
Patient lived …	Alone	175	21.1%	47	12.7%	31	20.9%	26	23.0%	71	35.9%	0.001*
	With 1-2 people	306	36.9%	132	35.7%	58	39.2%	52	46.0%	64	32.3%	
	With 3 people	210	25.3%	116	31.4%	35	23.6%	19	16.8%	40	20.2%	
	With 4 or more people	138	16.6%	75	20.3%	24	16.2%	16	14.2%	23	11.6%	
Having care for…	None	612	73.8%	296	80.0%	103	69.6%	70	61.9%	143	72.2%	0.001*
	Someone	217	26.2%	74	20.0%	45	30.4%	43	38.1%	55	27.8%	
Patient infected	No	769	92.8%	359	97.0%	147	99.3%	99	87.6%	164	82.8%	0.001*
	Yes	60	7.2%	11	3.0%	1	0.7%	14	12.4%	34	17.2%	
Closed one infected	No	703	84.8%	317	85.7%	132	89.2%	92	81.4%	162	81.8%	0.187
	Yes	126	15.2%	53	14.3%	16	10.8%	21	18.6%	36	18.2%	
Working	No	383	46.2%	179	48.4%	63	42.6%	59	52.2%	82	41.4%	0.175
	Yes	446	53.8%	191	51.6%	85	57.4%	54	47.8%	116	58.6%	
Financial loses	No	609	73.5%	298	80.5%	113	76.4%	70	61.9%	128	64.6%	0.001*
	Yes	220	26.5%	72	19.5%	35	23.6%	43	38.1%	70	35.4%	

		Young/Adol. *N=172*		Adults *N=657*		*p*	Europe *N=676*		Asia *N=153*		*p*
Age (yrs-old); *mean - SD*		15.66	1.25	31.10	11.90	0.001*	28.95	13.01	23.26	6.98	0.001*
Sex; *n* - %	Female	157	91.3%	427	65.0%	0.001*	438	64.8%	146	95.4%	0.001*
	Male	15	8.7%	230	35.0%		238	35.2%	7	4.6%	
Contextual lock-down		*n*	%	*n*	%	*p*	*n*	%	*n*	%	*p*
Patient lived …	Alone	5	2.9%	170	25.9%	0.001*	151	22.3%	24	15.7%	0.250
	With 1-2 people	55	32.0%	251	38.2%		242	35.8%	64	41.8%	
	With 3 people	70	40.7%	140	21.3%		169	25.0%	41	26.8%	
	With 4 or more people	42	24.4%	96	14.6%		114	16.9%	24	15.7%	
Having care for…	None	157	91.3%	455	69.3%	0.001*	543	80.3%	69	45.1%	0.001*
	Someone	15	8.7%	202	30.7%		133	19.7%	84	54.9%	
Patient infected	No	171	99.4%	598	91.0%	0.001*	616	91.1%	153	100.0%	0.001*
	Yes	1	0.6%	59	9.0%		60	8.9%	0	0.0%	
Closed one infected	No	139	80.8%	564	85.8%	0.102	553	81.8%	150	98.0%	0.001*
	Yes	33	19.2%	93	14.2%		123	18.2%	3	2.0%	
Working	No	80	46.5%	303	46.1%	0.927	324	47.9%	59	38.6%	0.036*
	Yes	92	53.5%	354	53.9%		352	52.1%	94	61.4%	
Financial loses	No	152	88.4%	457	69.6%	0.001*	493	72.9%	116	75.8%	0.465
	Yes	20	11.6%	200	30.4%		183	27.1%	37	24.2%	

Note. Adol: Adolescents. BED: Binge eating disorder. OSFED: other specified feeding eating disorder. SD: standard deviation. "*": significant comparison.

COVID Isolation Eating Scale- CIES

EFFECTS OF CONFINEMENT IN PATIENTS WITH EATING DISORDERS/OBESITY AND EVALUATION OF REMOTE INTERVENTIONS

The present scale aims to examine what effects confinement due to the COVID-19 pandemic has had in patients with eating disorders and/or obesity receiving treatment. The scale examines changes in eating symptoms and in other areas of interest relating to confinement. It also includes an evaluation of received remotely delivered interventions during confinement. The scale is designed to be answered both in person and by phone/online.

Current data: ____ / ___ / _______	Date of birth: ____ / ___ / ________	Age (years): _________
Country of residence:	City of residence:	
Sex: ⓪ Female ① Male ② Other	Height (m): _______________	
Weight before confinement: _____________ (Kg)	Current weight ________ (Kg)	
Confinement in my country was: ⓪ Voluntary ① Mandatory		
If applicable, date when total confinement ended in your place of residence: ____ / ___ / 2020		

I CIRCUMSTANCES DURING CONFINEMENT

1. If applicable, when did confinement for the COVID-19 pandemic begin in your case? ___ / ____ / _____

2. Who did you live with during confinement? (Check all possible options)..
   - ☐ I've been alone
   - ☐ Spouse / partner
   - ☐ Children
   - ☐ Father / mother
   - ☐ Father-in-law / mother-in-law
   - ☐ Other people (friends, ...)

   Indicate how many people you lived with during confinement: ___________

3. Have you had relatives or other people in your care during confinement? (Check all possible options) ....
   - ☐ I have not had anyone
   - ☐ Spouse / regular partner
   - ☐ Children
   - ☐ Father / mother
   - ☐ Father-in-law / mother-in-law
   - ☐ Other people (friends, ...)

   Indicate how many people you have had in your care during confinement: ___________

4. Did you contract COVID-19 during this period?.. ⓪ No ① Yes

   Has a family member or person close to you contract COVID-19 during this period? .... ⓪ No ① Yes

   If yes, indicate who: _____________

5. Have you cared for a family member or person infected with COVID-19 during this period?... ⓪ No ① Yes

   If yes, indicate who: _____________

6. If you were employed, have you continued to work during confinement?.. ⓪ No ① Yes ② Not Applicable

   If you have worked during confinement indicate how:
   - ① Remotely
   - ② In person
   - ③ Both

7. In your case, did confinement involve a partial or complete loss of economic income? ⓪ No ① Yes

8. Just before confinement, were you linked to treatment for an eating disorder and/or obesity?..
   - ⓪ No
   - ① Outpatient
   - ② Day-hospital
   - ③ Inpatient

   If receiving treatment, indicate since when (in months): ___________

II EFFECTS OF CONFINEMENT ON EATING SYMPTOMS

1. If applicable, indicate what is your current diagnosis of eating disorder:
   ① Anorexia nervosa ② Bulimia nervosa ③ Binge eating disorder ④ Not specified 5 Not Applicable

2. Have you been diagnosed with any other psychiatric disorder? (Check all possible options)

☐ None	☐ Specific phobia	☐ Substance abuse-dependence
☐ Anxiety	☐ Social phobia	☐ Behavioral addiction
☐ Depression	☐ Somatoform	☐ Psychosis
☐ Bipolar	☐ ADHD	☐ Impulse control
☐ Obsessive-compulsive	☐ Neurological	☐ Other / s ________________________

3. Do you have diabetes mellitus? ⓪ No ① Yes

   If yes, indicate whether during confinement

   a. You had difficulty following diet instructions.. ⓪ No ① Yes

   b. You have had a hard time obtaining your regular medication.. ⓪ No ① Yes

   c. In case of capillary blood glucose control, did your levels increase? .... ⓪ No ① Yes

   d. If consuming oral hypoglycemic medication, did you have to increase your dose? .... ⓪ No ① Yes

   e. If you injected insulin, did you have to increase your dose or the number of injections a day?... ⓪ No ① Yes

We are interested in exploring the extent to which each you have experienced the following behaviors, before and after confinement. For each behavior, check the box that best corresponds to your experience using this scale:

	Before confinement					Currently				
	Never	Hardly ever	Sometimes	Almost always	Always	Never	Hardly ever	Sometimes	Almost always	Always
4. Concern about weight, diet and body image	⓪	①	②	③	④	⓪	①	②	③	④
5. Deliberate restriction of the amount of food eaten	⓪	①	②	③	④	⓪	①	②	③	④
6. Deliberate restriction of the number of meals per day	⓪	①	②	③	④	⓪	①	②	③	④
7. Eat repeatedly in small amounts between meals throughout the day	⓪	①	②	③	④	⓪	①	②	③	④
8. Eating a large amount of food in a short period of time with feelings of loss of control (binge eating)	⓪	①	②	③	④	⓪	①	②	③	④
9. Induced Vomiting	⓪	①	②	③	④	⓪	①	②	③	④
10. Use of laxatives	⓪	①	②	③	④	⓪	①	②	③	④
11. Use of diuretics	⓪	①	②	③	④	⓪	①	②	③	④
12. Physical exercise with daily frequency	⓪	①	②	③	④	⓪	①	②	③	④
13. Constant performance of other activities involving physically spending energy	⓪	①	②	③	④	⓪	①	②	③	④

III REACTION TO CONFINEMENT

We are interested in exploring to what extent you experienced the behaviors mentioned below, before and after confinement. Check the box that best corresponds to your experience using this scale.

Never	Hardly ever	Sometimes	Almost always	Always
0	1	2	3	4

	Before confinement					Currently				
	Never	Hardly ever	Sometimes	Almost always	Always	Never	Hardly ever	Sometimes	Almost always	Always
1. Eat constantly throughout the day without realizing what is happening	⓪	①	②	③	④	⓪	①	②	③	④
2. Not being able to resist eating continuously between meals, despite trying not to	⓪	①	②	③	④	⓪	①	②	③	④
3. Eat when negative emotions are experienced such as: fear, anxiety, sadness, loneliness, anger or boredom	⓪	①	②	③	④	⓪	①	②	③	④
4. Feeling upset about eating constantly throughout the day	⓪	①	②	③	④	⓪	①	②	③	④
5. Deliberately eat weight loss foods	⓪	①	②	③	④	⓪	①	②	③	④
6. Have an intense urge to eat certain foods, after reducing or giving them up	⓪	①	②	③	④	⓪	①	②	③	④
7. Make an extra effort to get certain foods when they have not been available	⓪	①	②	③	④	⓪	①	②	③	④
8. Feeling irritable, nervous or sad when cutting down on or giving up certain foods	⓪	①	②	③	④	⓪	①	②	③	④
9. You need to eat more and more to reach the feeling you want to reach by eating. This includes, reducing negative emotions (such as sadness) or increasing positive emotions (such as pleasure).	⓪	①	②	③	④	⓪	①	②	③	④
10. Failing to cut down on or give up eating certain foods	⓪	①	②	③	④	⓪	①	②	③	④
11. Family members and/or partner dieting or carrying out other behavior aimed at losing weight	⓪	①	②	③	④	⓪	①	②	③	④
12. Difficulty falling asleep or insomnia	⓪	①	②	③	④	⓪	①	②	③	④
13. Hopeless or distressing thoughts about the current and future situation	⓪	①	②	③	④	⓪	①	②	③	④
14. Constant crying episodes	⓪	①	②	③	④	⓪	①	②	③	④

	Before confinement					Currently				
	Never	Hardly ever	Sometimes	Almost always	Always	Never	Hardly ever	Sometimes	Almost always	Always
15. Feelings of loneliness	⓪	①	②	③	④	⓪	①	②	③	④
16. Agitation or nervousness	⓪	①	②	③	④	⓪	①	②	③	④
17. Lack of willingness to maintain contact with family or friends	⓪	①	②	③	④	⓪	①	②	③	④
18. Intrusive thoughts related to death	⓪	①	②	③	④	⓪	①	②	③	④
19. Loss of desire or sexual pleasure	⓪	①	②	③	④	⓪	①	②	③	④
20. Extreme concern over health and the risk of infectious disease contagion and/or COVID19	⓪	①	②	③	④	⓪	①	②	③	④
21. Anxiety and nervousness related to infectious disease and/or COVID19	⓪	①	②	③	④	⓪	①	②	③	④
22. Concern regarding loss of employment and / or income	⓪	①	②	③	④	⓪	①	②	③	④
23. Feeling of out-of-control emotions	⓪	①	②	③	④	⓪	①	②	③	④
24. Difficulty understanding feelings	⓪	①	②	③	④	⓪	①	②	③	④
25. Feelings of anger or shame when having negative emotions	⓪	①	②	③	④	⓪	①	②	③	④
26. Aggressive behavior towards oneself, objects or other people	⓪	①	②	③	④	⓪	①	②	③	④
27. Irritability and / or constant discussions	⓪	①	②	③	④	⓪	①	②	③	④
28. Tobacco use	⓪	①	②	③	④	⓪	①	②	③	④
29. Alcohol consumption	⓪	①	②	③	④	⓪	①	②	③	④
30. Consumption of other psychoactive substances	⓪	①	②	③	④	⓪	①	②	③	④
31. Make online purchases of products that are not essential and feeling that this is out of control	⓪	①	②	③	④	⓪	①	②	③	④
32. Use of video games or games and / or online gambling	⓪	①	②	③	④	⓪	①	②	③	④
33. Spend a large part of the day using social media and / or the internet (not for academic or work purposes)	⓪	①	②	③	④	⓪	①	②	③	④
34. Have a problem with gambling	⓪	①	②	③	④	⓪	①	②	③	④

IV EVALUATION OF REMOTE INTERVENTIONS

Please check the corresponding box considering your degree of agreement / disagreement with the following statements using this scale:

Totally disagree	Disagree	Neither agree nor disagree	Agree	Totally agree
0	1	2	3	4

	Totally disagree	Disagree	Neutral	Agree	Totally agree
1. I believe that the remote therapy received during confinement has been adequate	⓪	①	②	③	④
2. The transition to remote therapy was easy for me	⓪	①	②	③	④
3. I have felt comfortable with the new intervention	⓪	①	②	③	④
4. I am motivated to continue with telematic therapy	⓪	①	②	③	④
5. I feel confident of the confidentiality in the new intervention	⓪	①	②	③	④
6. I consider the interaction with the psychologist during the remote intervention to be satisfactory	⓪	①	②	③	④
7. The interaction with the psychologist was similar to that of the face-to-face sessions	⓪	①	②	③	④
8. In general, I am satisfied with the remote intervention I received	⓪	①	②	③	④
9. I am willing to continue receiving remote therapy as long as necessary	⓪	①	②	③	④
10. I think the new treatment has been effective during confinement	⓪	①	②	③	④

11. What do you consider to be the main challenges you faced with remote therapy during confinement?

12. What do you consider having been the main strengths of the remote intervention during confinement?

13. What do you consider to be the main weaknesses of the remote intervention during confinement?
